# A structural model of treatment program and individual counselor leadership in innovation transfer

**DOI:** 10.1186/s12913-017-2170-y

**Published:** 2017-03-23

**Authors:** George W. Joe, Jennifer E. Becan, Danica K. Knight, Patrick M. Flynn

**Affiliations:** Institute of Behavioral Research, Texas Christian University, TCU Box 298740, Fort Worth, TX 76129 USA

**Keywords:** Innovation transfer, Leadership, Staff attributes, Change agents, Drug treatment programs

## Abstract

**Background:**

A number of program-level and counselor-level factors are known to impact the adoption of treatment innovations. While program leadership is considered a primary factor, the importance of leadership among clinical staff to innovation transfer is less known. Objectives included explore (1) the influence of two leadership roles, program director and individual counselor, on recent training activity and (2) the relationship of counselor attributes on training endorsement.

**Methods:**

The sample included 301 clinical staff in 49 treatment programs. A structural equation model was evaluated for key hypothesized relationships between exogenous and endogenous variables related to the two leadership roles.

**Results:**

The importance of organizational leadership, climate, and counselor attributes (particularly counseling innovation interest and influence) to recent training activity was supported. In a subset of 68 counselors who attended a developer-led training on a new intervention, it was found that training endorsement was higher among those with high innovation interest and influence.

**Conclusions:**

The findings suggest that each leadership level impacts the organization in different ways, yet both can promote or impede technology transfer.

**Electronic supplementary material:**

The online version of this article (doi:10.1186/s12913-017-2170-y) contains supplementary material, which is available to authorized users.

## Background

Because each organizational environment represents a complex human activity system that directly impacts technology/innovation transfer, studies of innovation transfer within drug treatment programs can benefit from understanding its organizational factors [1]. Organizational [2, 3], systemic (e.g., workplace stress perceptions), and idiographic variables (e.g., longer service tenure [4]) have been shown to predict implementation and adoption attitudes.

Many frameworks and models have been proposed for understanding this issue [5–10]. One literature review of 81 studies on quantitative and qualitative factors affecting implementation [11] identified five essential areas (innovations, providers, communities, organizational capacity, training/technical assistance) with conclusions summarized in a hierarchical framework consisting of three nested ovals. Adaptability of the innovation in the proposed environment is central among innovation characteristics addressed in the innermost oval [7, 11] where implementation is hypothesized to be a function of organizational capacity (e.g., leadership, a program champion/internal advocate, managerial/supervisory support) and training/technical assistance. The middle oval encompassed provider characteristics (e.g., perceived need and perceived benefits of the innovation, self-efficacy, and skill proficiency for the innovation). The outer oval consists of community factors (e.g., funding, community readiness for change). The emphasis on training and technical assistance as central to improvement efforts is one of the review’s important contributions.

Factors affecting organizational capacity include a positive work climate, organizational norms regarding change, new programming integration, and shared vision, while social context/work environment perceptions affect not only technical assistance usage [5, 8, 12, 13] but also adoption, implementation, sustainment and effectiveness [8]. Another relevant organizational process factor is absorptive capacity [14]. This addresses access and ability to use information effectively by the organization [15, 16]. It is enhanced by a professional workforce, engagement in environmental scanning (i.e., identification of training and professional development opportunities), and satisfaction data from the organization’s buyers and suppliers. Moreover, identification of individuals who serve as “information brokers” can enhance absorptive capacity.

While physical and perceived program resources have been shown to affect training exposure and utilization [17], it is program leadership that is considered the primary factor, as it affects the entire organizational environment and is instrumental in adoption decisions [18]. Strong leadership is particularly important to organizational climate in times of system and organizational change [19] by making the climate more open to change and by empowering staff to be participants in change [20]. Delegation of authority to competent people is important [21], for they can serve as effective change agents through working/bonding with treatment teams, administrators, key opinion leaders, and community stakeholders [6, 8, 22]. Valued characteristics for internal change agents include self-efficacy [23–25] and adaptability [9, 26].

Similarly, interest in innovations and influence with other staff are likely important attributes [27, 28], as are growth, efficacy, influence, and adaptability. Being employed in organizational climates conducive to change (i.e., higher mission, cohesiveness, autonomy, communication, and openness to change, but lower stress) have been found to have more favorable attitudes about innovation training and its adoption [28]. Professional growth and influence predicts openness to the use of medications [27] while staff adaptability and influence significantly predicts opinions toward treatment manuals and integrated mental health services [27]. Thus, implementation of new practices is influenced directly by program leadership’s adoption decisions and indirectly through leadership’s shaping of the organizational climate, task delegation, and selection of change agents [1, 20].

### Current study model

**Fig. 1:**
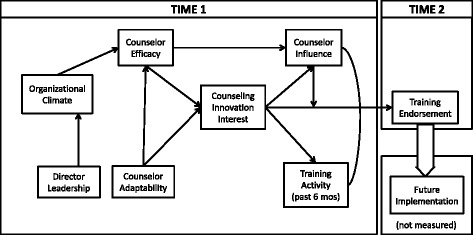
Conceptual model of counseling innovation interest on training participation and endorsements

Of the many factors that have been proposed for better understanding innovation transfer, increased insight into the interrelationships among program leadership, organizational climate, and change agents would seem to be critical. Furthering knowledge about these change agents to whom transfer tasks are delegated with regard to what might be potential effective characteristics and their relationship to program leadership and climate is an area that requires additional study. For the current study, the proposed interrelationships of selected factors related to this concept are examined simultaneously in an implementation model for drug treatment innovations. The interrelated roles among program leadership, organizational environment, counselor leadership, and the clinical staff attributes are conceptualized in the path model presented in Fig. 1 and summarized by the hypotheses below. Principal are the effects of two leadership paths (director and staff).

Hypotheses

1. Director Leadership will have direct effects on Organizational Climate (OC).
2. Director Leadership will have an indirect positive relationship with Counseling Innovation Interest (CII) through its relationships with OC and Counselor Efficacy, which has a direct positive relationship with CII.
3. Counselor Adaptability will have both direct and indirect (through Counselor Efficacy) positive relationships with CII.
4. CII will be positively related to Counselor Influence and participation in recent trainings.
5. Counselor Influence will moderate the relationship between CII and subsequent endorsements of innovation adoption following training.

## Methods

### Procedure

Data were collected in 2013 as part of the Treatment Readiness and Induction Program (TRIP) project funded by the National Institute on Drug Abuse (NIDA), National Institutes of Health (NIH), and Department of Health and Human Services. The project was structured as 2 phases. Effectiveness was examined in Phase 1 [29, 30]. Phase 2 examined implementation of TRIP in multiple juvenile-justice and community-based sites in the United States.

The current study is based on the phase 2 data. Regional Addiction Technology Transfer Centers (ATTCs), which are responsible for providing training and technical assistance to agencies and their staffs on best practices for substance use, assisted with program recruitment. With the help of four ATTCs (Great Lakes, South Southwest, Pacific Southwest, and Northeast and Caribbean), the implementation sample included 312 counselors from 52 adolescent treatment facilities located in 12 US states. Institutional Review Boards (IRBs) for the research center and treatment programs reviewed and approved study protocols.

Data collection procedures consisted of (a) pre-training survey of the organization (completed by director or director’s designee), (b) pre-training survey of counseling staff, (c) survey at the conclusion of the training by “trainees,” and (d) follow-up counseling staff survey at 4 months after the training.

Pre-training data were collected from February to July 2013. About 1–2 months prior to the training workshop, all counseling staff at the participating facilities with direct client contact (counselors, social workers, case managers, clinical supervisors, and therapists) were provided with information describing the study and informed consent was obtained. Participants were asked to complete the TCU Survey of Organizational Functioning and Leadership (SOFL; [31]), which as noted by Broome and his colleagues is the TCU Survey of Organizational Functioning (SOF) and 4 subscales (Encourages Innovation, Inspirational Motivation, Develops Others, Task Delegation) from the Survey of Transformational Leadership instrument [32]. The SOFL scales covered organizational climate, staff attributes, motivation for change, resources, job attitudes, workplace practices, transformational leadership, and training exposure and utilization. Additionally, one month prior to the training, the program director (or program designee) completed the TCU Survey of Structure and Operations (SSO) [33], which included organizational level information.

Approximately 1 month after staff surveys were administered, 1–2 clinical staff at each participating program were chosen by its program director to attend a 2-day developer-led workshop on use of the TRIP curriculum and serve as trainers. At the conclusion of the 2-day workshop (approximately 13 h), participants received a survey consent form and were asked to complete a 30-min TCU Workshop Evaluation Form (WEVAL) [34] addressing satisfaction with the workshop/materials, perceived adequacy of resources, training, and support for implementing TRIP at their treatment program. These data were collected anonymously onsite by research staff.

### Participant samples

Two samples were examined in the current study: (a) counseling staff who completed the SOFL at Time 1 (used to examine the structural model), and (b) counseling staff trainees (used to examine the interaction of counselor innovation interest with attitudes on subsequent endorsement of TRIP). There were 312 counselors from 52 programs who completed the SOFL. Because of missing data on analysis variables, 11 staff representing 3 facilities were omitted. From the SSO, the counselors were classified into 6 treatment modality groups: secure juvenile justice settings (SJJ), residential or modified therapeutic community with 60% or more juvenile justice clients (R/TC + 60), other residential or modified therapeutic community (R/TC-60), outpatient with 60% or more juvenile justice clients (OP + 60), other outpatient (OP-60), or both outpatient and residential/modified therapeutic community (OP/TC). This was done to adjust for possible modality differences that might affect the results. The final sample for examining the structural model (hypotheses 1–4) included 301 clinical staff, representing 49 facilities (7 SJJ, 7 R/TC + 60, 13 R/TC-60, 5 OP + 60, 12 OP-60, and 5 OP/TC).

There were 75 counselors from 47 treatment programs who attended TRIP training and completed the WEVAL [34]. Of these clinical trainees, 68 had data on the WEVAL scales and the SOFL measure on counselor innovation interest that were used for examining post-training attitudes in hypothesis 5. They represented 43 treatment facilities [7 SJJ: 12 staff; 6 R/TC + 60: 10 staff; 9 R/TC-60: 12 staff; 11 OP-60: 19 staff; 5 OP + 60: 9 staff; 5 OP/TC: 6 staff]. Table 1 presents background information on both the sample used for the structural model and the subsample of clinical trainees. The table suggests there are similarities between the two samples. In both samples, the average age was approximately 41, the percentage of females was nearly two-thirds, Whites comprised a majority (although a smaller percentage in the TRIP training sample), and as expected, a majority had a bachelor’s degree or higher.

**Table 1:** Background characteristics of structural equation model and TRIP training samples

	Research samples			
	SEM Model Sample (SOFL)		TRIP Training (WEVAL)	
Background measures	N	Mean (SD)	N	Mean (SD)
Age	297	41.5 (12.5)	68	40.8 (10.7)
Male (%)	296	34.1%	68	32.4%
Race (%)	294		67	
African American	47	16.0%	9	13.4%
White	183	62.2%	37	55.2%
Hispanic	42	14.3%	14	20.9%
Mixed	11	3.7%	1	1.5%
Other	11	3.7%	6	9.0%
Degree	301			
Less than college	13	4.3%	4	5.9%
Some college	37	12.3%	5	7.4%
Associate’s degree	21	7.0%	4	5.9%
Bachelor’s degree	74	24.6%	11	16.2%
Master’s degree	141	46.8%	38	55.9%
Higher degree	15	5.0%	6	8.8%
Addiction Certification Status	290		68	
Not certified	130	44.8%	21	30.9%
Previously certified	5	1.7%	3	4.4%
Currently certified	125	43.1%	35	51.5%
Intern	30	10.3%	9	13.2%
Years Counseling	298			
< 6 months	50	16.8%	5	7.4%
6–11 months	12	4.0%	3	4.4%
1–3 years	68	22.8%	13	19.1%
3–5 years	48	16.1%	15	22.1%
> 5 years	120	40.3%	32	47.1%
Current job tenure	298			
< 6 months	46	15.4%	11	16.7%
6–11 months	34	11.4%	8	12.1%
1–3 years	86	28.9%	18	27.3%
3–5 years	41	13.8%	11	16.7%
> 5 years	91	30.5%	18	27.3%
Discipline/Profession	301			
Addiction counseling	183	60.8%	46	67.6%
Social work	113	37.5%	28	41.2%
Other counseling	96	31.9%	19	27.9%
Psychology	66	21.9%	19	27.9%
Criminal justice	28	9.3%	11	16.2%
Administration	29	9.6%	6	8.8%
Education	26	8.6%	6	8.8%
Vocational Rehab	10	3.3%	3	4.4%
Nurse	5	1.7%	0	NA
Medicine other	4	1.3%	0	NA
Other	34	11.3%	6	8.8%

### Measures

#### Organizational climate, staff attributes, and director leadership

The SOFL [31, 32] is a 165-item instrument. The SOFL includes the TCU Organizational Readiness for Change (ORC) instrument, which measures 18 dimensions covering four major areas: Program Needs/Pressures, Program Resources, Staff Attributes, and Organizational Climate [35]. The background and development of these scales have been discussed previously, emphasizing their intended link to organizational change [35]. In addition to the ORC, the SOFL includes 8 additional scales covering workplace practices and job attitudes as well as 4 scales from the TCU Survey of Transformational Leadership (STL) instrument: encourages innovation, inspirational motivation, develops others, and task delegation [32]. Each item was rated on a 5-point Likert scale [1 = disagree strongly, 2 = disagree, 3 = uncertain, 4 = agree, 5 = agree strongly; 32, 31]. In computing the scale scores, the responses on the items for a scale are averaged and then multiplied by 10. The present study uses items from the staff attributes, organizational climate, and transformational leadership scales to address the hypotheses. These scales are listed in Table 2.

**Table 2:** Scales and sample items included in estimated structural model

	N	Mean (SD)
Transformational Leadership (Edwards, Knight, Broome, & Flynn, 2010)	297	37.9 (7.4)
Encourages Innovation (4 items; α = .87):	297	39.1 (7.7)
o Program director positively acknowleges creative solutions to problems.		
o Program director encourages ideas other than own.		
Inspirational Motivation (6 items; α = .91):	297	38.7 (7.4)
o Program director displays enthusiasm about pursuing program goals.		
o Program director expresses confidence in staff members’ collective ability to reach program goals.		
Develops Others (4 items; α = .88):	297	36.5 (8.6)
o Program director offers individual learning opportunities to staff members for professional growth.		
o Program director takes into account individual abilities when teaching staff members.		
Task Delegation (9 items; α = .94):	297	37.4 (7.8)
o Program director follows delegation of a task with support and encouragement.		
o Program director allocates adequate resources to see tasks are completed.		
Organizational Climate (Lehman, Greener, & Simpson, 2002)	297	33.6 (5.5)
Clarity of Mission (5 items; α = .73):	297	35.0 (6.5)
o Duties clearly related to the goals of this program.		
o This program operates with clear goals and objectives.		
Staff Cohesiveness (6 items; α = .88):	297	36.2 (8.2)
o Staff here all get along very well.		
o Staff here are always quick to help one another when needed.		
Staff Autonomy (5 items; α = .56):	297	35.2 (5.1)
o Management here fully trusts your professional judgment.		
o Counselors here are given broad authority in treating their own clients.		
Communication (5 items; α = .81):	297	33.5 (7.3)
o Program staff are always kept well informed.		
o Formal and informal communication channels here work very well.		
Stress (4 items; α = .81):	297	32.6 (8.4)
o You are under too many pressures to do your job effectively.		
o The heavy workload here reduces program effectiveness.		
Openness to Change (5 items; α = .72):	297	34.3 (6.2)
o It is easy to change procedures here to meet new conditions.		
o You are encouraged here to try new and different techniques.		
Staff Attributes (Lehman, Greener, & Simpson, 2002)		
Efficacy (5 items; α = .68):	297	40.8 (4.9)
o You have skills needed to conduct effective group counseling.		
o You are effective and confident in doing your job.		
Influence (6 items; α = .80):		
Willingness and ability of a counselor to influence co-workers	297	37.5 (6.9)
o Staff generally regard you as a valuable source of information.		
o You are viewed as a leader by other staff here.		
Adaptability (4 items; α = .65):		
Staff ability to adapt to a changing environment	297	40.2 (5.2)
o Learning and using new procedures are easy for you.		
o You are able to adapt quickly when you have to shift focus.		
Counseling Innovation Interest (CII; 9 items; α = .85):	297	3.51 (.65)
• You read about new techniques and treatment information each month.		
• You regularly read professional journal articles or books on drug abuse treatment.		
• You do a good job of regularly updating and improving your skills.		
• When you attend workshops, how often do you try out the new interventions or techniques learned?		
• In recent years, how often have you adopted (for regular use) new counseling interventions?		
• You like to use new types of therapy/interventions to help your clients.		
• You are willing to try new types of therapy/interventions even if you have to follow a treatment manual.		
• You would try a new therapy/intervention even if it were very different from what you are used to doing.		
• You are willing to use new and different types of therapy/interventions developed by researchers.		

#### Recent training activity

Three items from the SOFL Training Exposure index addressed “active participation in training in last 6 months.” These included “learned new skills or techniques at a professional conference,” “how often attended training workshop within 50 miles,” and “how often attended training workshop over 50 miles.” The first item using the 5-point response format (disagree strongly/agree strongly) was dichotomized (disagree strongly, disagree, or uncertain = 0, agree or agree strongly = 1). The latter two items had a 5-point response format (1 = none, 2 = 1, 3 = 2, 4 = 3, and 5 = 4 or more) and they were also dichotomized (“none” = 0 or “at least 1” =1). This 3-item index had a coefficient alpha of .59.

#### Counseling innovation interest (CII)

This scale, counseling innovation interest, addressed both seeking professional growth and “openness to using new counseling technology” [36]. As an expression of innovation interest, it includes previous staff attendance at workshops [36] and growth-related personal activities, including willingness to try new therapeutic approaches [20]. This measure taps “leadership initiative” among counseling staff, paralleling the organizational absorptive capacity (identification of training and professional development ideas) at the individual level measure and may be indicative of an individual’s potential as an internal agent of change within the organization. It is hypothesized to serve as a mediator between counselor attributes (efficacy and adaptability) and indicators of implementation (e.g., positive training endorsement, adoption, use of new practices). The CII measure was created using 9 items (see Table 2) from the SOFL including reading about new treatment techniques, updating skills, and willingness to try new therapeutic approaches.

#### Training endorsements

The TCU Workshop Evaluation Form for TRIP Training (WEVAL) [34] consists of 42 items with a 5-point Likert response scale (1 = Disagree Strongly, 2 = Disagree, 3 = Uncertain, 4 = Agree, and 5 = Agree Strongly). Five scales (Table 3) were used and they addressed staff attitudes toward using TRIP within their treatment setting: Acceptability, Appropriateness, Adoption Expectation, Preparation Adequacy, and Leadership Engagement. Scores for each scale were calculated by averaging responses to its set of items and multiplying by 10.

**Table 3:** Scales and items from the TCU Workshop Evaluation Form (WEVAL)

	N	Mean (SD)
Acceptability (8 items; α = .68)	68	42.0 (3.0)
• You are satisfied with the materials in the TRIP curriculum.		
• TRIP seems cumbersome and difficult to use.®		
• TRIP materials seem easy to use.		
• There are too many steps involved in TRIP.®		
Appropriateness (7 items; α = .79)	68	42.3 (4.4)
• TRIP is relevant to the needs of your clients.		
• TRIP fits with your counseling style.		
• You already use materials similar to TRIP and see no reason to change.®		
• Your program has used similar materials in the past with little success.®		
• TRIP can be useful for addressing client motivation.		
• TRIP can be useful for addressing client participation.		
• TRIP can be useful for addressing client decision making.		
Adoption Expectation (1 item)	68	45.3 (6.3)
• You expect the things you learned in this workshop will be used in your program within the next month or so.		
Preparation Adequacy (5 items; α = .71)	68	42.3 (4.2)
• You are comfortable using TRIP materials with your clients.		
• You feel properly prepared to use TRIP.		
• You feel able to train others to conduct TRIP.		
• Staff at your program will want to start their own TRIP groups when they see the materials.		
• You will encourage clients to attend TRIP groups once they are offered.		
Leadership Engagement (5 items; α = .71)	68	38.6 (5.1)
• Your program leaders encourage staff to conduct TRIP groups.		
• Leadership at your program provides resources for innovations, like TRIP.		
• Your program leadership places adoption of TRIP as a priority.		
• Leadership within your agency encourages staff to use TRIP materials within their regular sessions.		
• Leadership within your agency recognizes staff that use new approaches, such as TRIP.		

® Item reversed scored

### Analytic plan

In order to account for treatment modality differences, a within-modality covariance matrix was used for the analysis of the structural model. This estimated matrix represents the covariance matrix common to the treatment modalities. It was obtained from SAS PROC DISCRIM [37], and the structural equation model was tested using SAS PROC CALIS [37]. Goodness-of-fit *χ* ^2^, normed *χ* ^2^, root mean square error of approximation (RMSEA), standardized root mean square residual (SRMSR), comparative fit index (CFI), and Normed fit index (NFI) were used to assess the fit of the model to the data. For goodness-of-fit *χ* ^2^, RMSEA, and SRMSR, smaller values indicate better fits. Because the goodness of fit *χ* ^2^ may be sensitive to sample sizes larger than 200, the normed *χ* ^2^ (*χ* ^2^ divided by its degrees of freedom) is considered a better *χ* ^2^ indicator of fit; that is, when less than 3.84 [38]. For RMSEA, values of .01, .05, and .08 indicate excellent, good, and mediocre fit, respectively [39]. For SRMSR, a value less than .08 is considered a good fit [40]. The CFI ranges from 0 to 1, with values above 0.9 indicating reasonable fit. For NFI, values of .90 to .95 reflect a good model fit. For hypothesis 5, SAS PROC GLM was used to address endorsement in terms of CII and Influence.

## Results

### Hypotheses 1–4: model for counseling innovation interest (CII)

#### Initial model

The initial structural model examined the first 4 hypotheses of the conceptual model outlined in Fig. 1 and was based on the sample of 301 counselors. While all estimated paths were significant, with the exception of the path from CII to Influence, this “basic model” (Time 1 measures only; excluding Training Endorsements and Future Implementation) had relatively “poor fit-statistics” [*χ* ^2^(11) = 44.6993, *p* < .0001; normed *χ* ^2^ = 4.06; RMSEA = .102; SRMSR = .072; CFI = .94; NFI = .92].

#### Revised model

**Fig. 2:**
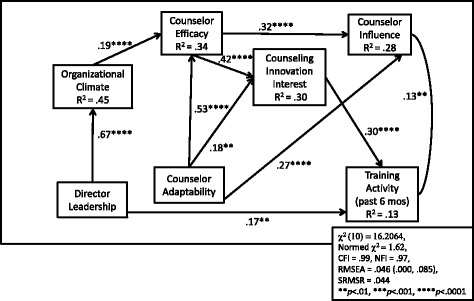
Estimated structural model for leadership, climate, staff attributes, counseling innovation interest, and training activity relationships

An examination of the Lagrange Multiplier test indices suggested two modifications: a path from Counselor Adaptability to Counselor Influence and a path from Director Leadership to Recent Training Activity. These modifications were made, as well as the deletion of the path from CII to Influence, and this revised model was re-estimated. Results indicated a “good fit” since its RMSEA was under .05. All other fit criteria were also improved [*χ* ^2^(10) = 16.2064, *p* < .0939; normed *χ* ^2^ = 1.62; RMSEA = .046 (.000, .085); SRMSR = .044; CFI = .99; NFI = .97], and all hypothesized paths were significant at *p* < .01 or less. This final model is displayed in Fig. 2.

The significant paths (standardized) were from Director Leadership to Organizational Climate (*b* = .67, *se* = .03, *t* = 20.84, *p* < .0001) and to Recent Training Activity (*b* = .17, *se* = .05, *t* = 3.21, *p* < .0013). From Counselor Adaptability there were significant paths to Counselor Efficacy (*b* = .53, *se* = .04, *t* = 13.01, *p* < .0001), CII (*b* = .18, *se* = .06, *t* = 3.13, *p* < .002), and Counselor Influence (*b* = .27, *se* = .06, *t* = 4.78, *p* < .0001). Organizational Climate had a significant path to Counselor Efficacy (*b* = .19, *se* = .05, *t* = 3.99, *p* < .0001). Counselor Efficacy had significant paths to CII (*b* = .42, *se* = .05, *t* = 7.68, *p* < .0001) and Counselor Influence (*b* = .32, *se* = .06, *t* = 5.71, *p* < .0001). CII had a significant path to Recent Training Activity (*b* = .30, *se* = .05, *t* = 5.86, *p* < .0001). Counselor Influence was significantly correlated with Training Activity (*r* = .13, *se* = .05 *t* = 2.90, *p* < .004).

Results suggested that both Counselor Efficacy and CII are pivotal endogenous variables. Counselor Efficacy carried an indirect path to CII and Counselor Influence through its relationship with Organizational Climate and Counselor Adaptability. CII was a key predictor of Recent Training Activity in the model but not Counselor Influence. The R^2^ values (which represent the proportions of variance that the predictors account for in each endogenous variable) ranged from .13 (Recent Training Activity) to .45 (Organizational Climate). The R^2^ for the two key endogenous variables (Counselor Efficacy and CII) were .34 and .30, respectively, indicating these two variables had fairly large percentages of variance being accounted for.

### Hypothesis 5: effects of CII and counselor influence on training endorsement

While a fairly large sample was available to test the structural model, only the subset of 68 counselors selected to attend training on implementing TRIP were available to address endorsement. This subsample therefore does not represent a random sample of the total as they were selected by their treatment sites to attend the training. It might be expected that these counselors would be higher on some of the characteristics that are considered important in technology transfer compared to the remaining sample.

Indeed, it was found that the subsample was significantly higher on Counselor Influence [TRIP Training: mean = 40.0 (SD = 5.8); non-TRIP Training: mean = 36.8 (SD = 6.9), *t* = 3.36, *p* < .0009] and on Counselor Adaptability [TRIP Training: mean = 41.3 (SD = 4.4); non-TRIP Training: mean = 39.8 (SD = 5.3), t = 2.00, *p* < .046]. However, the two samples were not significantly different on Counselor Efficacy, Organizational Climate, Director Leadership, CII, or Recent Training Activity.

Even though the trainees were found to be higher on some of the characteristics that are considered important in technology transfer (Counselor Influence, Counselor Adaptability), it was expected that attribute variations existed within this trainee subsample and that level of counselor influence would moderate the relationship between CII and endorsement of innovation adoption. To display the relationships between training endorsement measures with Influence and CII (hypothesis 5), both Influence and CII were dichotomized at 35 or higher (agreement range) versus lower than 35 (disagreement range).

**Fig. 3:**
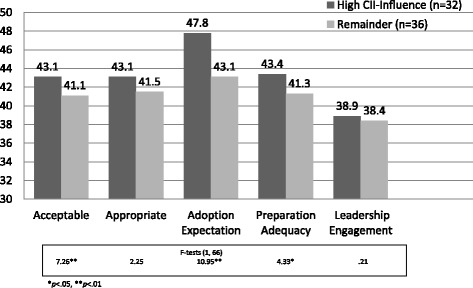
Future training (WEVAL) outcomes by CII and influence groups

Of the four quadrants produced by these two dichotomous variables (high CII and high Influence; high CII but low Influence; low CII but high Influence; low CII and low Influence), two quadrants contained very few individuals [4 in the “both low” quadrant (5.9% of total); 6 in the high CII but low Counselor Influence quadrant (8.8% of total)]. Therefore, to test the “interaction hypothesis,” the individuals who possessed both high CII and Counselor Influence (*n* = 32) were compared with all other individuals (*n* = 36). It was hypothesized that the group who had high scores on both measures would show significantly greater workshop endorsements relating to Acceptability of workshop training, Appropriateness of TRIP for their setting, Adoption Expectations, and Preparation Adequacy. As shown in Fig. 3, the high CII-Influence group had significantly higher means than the remainder group for Acceptability {(43.1 vs. 41.1); [F(1, 66) = 7.26, *p* < .01, η^2^ = .10, ES = .33]}, Adoption Expectation {(47.8 vs. 43.1); [F(1, 66) = 10.95, *p* < .0009, η^2^ = .14, ES = .40]}, and Preparation Adequacy {(43.4 vs. 41.3); [F(1, 67) = 4.33, *p* < .041, η^2^ = .06, ES = .25]}. The two groups were not significantly different on Leadership Engagement {(38.9 vs. 38.4); [F(1, 66) = .21, *p* < .65, η^2^ = .00, ES = .00]} or Appropriateness {(43.1 vs. 41.5); [F(1, 67) = 2.25, *p* < .14, η^2^ = .03, ES = .18]}. The significant findings were associated with medium to large effect sizes [41].

## Discussion

The present work is an exploratory study examining how “leadership” is important to the facilitation of implementation. It extends the work of Becan and colleagues [20] by conceptualizing change-oriented staff attributes (efficacy, adaptability, influence and professional growth) as “opinion leadership” and modeling the interrelationships of program leadership, organizational climate, opinion leadership and participation in recent trainings.

Two avenues of leadership and their potential effects on technology transfer were explored. The first was that overall leadership (represented by the director of the drug treatment organization) can affect implementation by instilling an organizational climate favorable to innovation adoption. The second was “opinion leadership” [6] among some counselors, as represented by an interest in innovation adoption and who have influence among co-workers within the organization. These counselors might serve as change agents, especially if they are delegated with some responsibility for orchestrating the new service implementation, and they might be candidates to become trainers in the train-the-trainer model for innovation implementation [42–45].

In summary, there were two key exogenous variables (variables not predicted by any other variable in the model) and five key endogenous variables (variables predicted by a variable in the model). The exogenous variables – Director Leadership and Counselor Adaptability – had significant paths to CII, either indirectly (Director Leadership) or directly (Counselor Adaptability). Director leadership had a pervasive relationship on all of the endogenous variables through its path to the organizational climate of the treatment program. Counselor Adaptability was related to multiple variables in the model directly, including perceptions of Counselor Efficacy, CII, and Counselor Influence. The relationship of Counselor Adaptability to Counselor Efficacy was stronger than Organizational Climate to Counselor Efficacy.

These findings corroborate previous work documenting the cascading effect that director leadership has on the organizational environment and subsequently on counseling staff attributes [20]. The findings also support the importance of both program leadership and organizational environment in implementing change [2, 14, 46].

While the present results were promising, they are not definitive, as the model data were limited and collected in a single instrument. Therefore, the results only confirm that the paths for the constructs are consistent with their hypothesized relationships but not evidence for causality. That would require additional data collection and testing. Nevertheless, the study does provide evidence that leadership both at the management level and at the staff level deserve further consideration in implementation efforts. The delegation of implementation activities to key staff who have an “aptitude” (e.g., innovation interest, efficacy, adaptability, and influence) for assisting in implementation, and who can serve as a “champion of the innovation” among staff, is believed to be critical to success. The results are consistent with previous research that show unique connections between director leadership, climate, and staff level leadership attributes, and contribute to previous work showing organizational climate as a mediator of the relationship between leadership and staff turnover intentions and turnover [19].

The second analysis addressed counselor-level leadership (high counseling innovation interest and individual influence) effects on endorsement attitudes toward treatment innovations. While this subsample reported higher Counselor Influence and Counselor Adaptability compared to the total sample, reported differences in training endorsement were detected. The results support the idea that counselors who pursue clinical skills development and who have influence among other staff are likely to have a skill set that positions them to champion change in their organization [2]. Future studies are needed to examine the process by which the combined effect of strong program leadership and the presence of change agents (staff that avidly pursue clinical skill development and influence coworkers in the use of new innovations) can together promote the uptake of innovations among staff within their agency.

While there are several strengths of this study, including the potential to generalize these findings to a wide variety of adolescent treatment settings, limitations should be acknowledged. First, data for the estimated model are cross-sectional, thereby eliminating the possibility to examine causality. Second, the sample to evaluate the effect of counselor innovation interest and influence among coworkers on innovation endorsement was small. Third, the examination of this combined effect was conducted on a specific innovation, which limits generalizability to other innovations.

## Conclusions

Leadership at the helm as well as within the ranks are important factors in technology transfer. Directors that involve staff in decision making and who delegate implementation tasks could bolster staff adaptability and self-identified influential staff. Key people leading change, supportive organizational culture, a positive pattern of managerial and clinical relations, and match between the core mechanisms of the innovation with the change agenda are important to successful change [47]. Absence of leadership at either level is a barrier to innovation adoption.

As noted by Flynn and Brown [48, 49], the knowledge base for guiding adoption and implementation of evidence-based practices (EBP) in the drug use treatment field is still modest. Treatment program resources are often limited, and programs are not likely to undertake change by themselves or allocate resources for an external change agent to guide implementation. Therefore, using internal personnel with “innovation leadership attributes” can help minimize costs associated with system change by serving as a resource in assisting implementation of certain manual-driven EBPs.

## Additional file

Additional file 1: Within groups covariance matrix used in modeling. Raw Data for subsample of counselors who did training. (DOC 123 kb)

## Abbreviations

ATTC: Addiction Technology Transfer Centers
CFI: Comparative fit index
CII: Counseling innovation interest
EBP: Evidence-based practices
IRB: Institutional Review Board
NFI: Normed fit index
NIDA: National Institute on Drug Abuse
NIH: National Institutes of Health
OC: Organizational Climate
ORC: Organizational Readiness for Change
RMSEA: Root mean square error of approximation
SJJ: Secure juvenile justice
SOF: Survey of Organizational Functioning
SOFL: Survey of Organizational Functioning and Leadership
SRMSR: Standardized root mean square residual
SSO: Survey of Structure and Operations
STL: Survey of Transformational Leadership
TRIP: Treatment Readiness and Induction Program
WEVAL: Workshop Evaluation Form
