# Supplementary material for: A structural model of treatment program and individual counselor leadership in innovation transfer
Source: BMC Health Serv Res. 2017 Mar 23;17:230. doi: 10.1186/s12913-017-2170-y (PMC5364669; doi:10.1186/s12913-017-2170-y)
Supplement: Additional file 1: — Within groups covariance matrix used in modeling. Raw Data for subsample of counselors who did training. (DOC 123 kb) [file 12913_2017_2170_MOESM1_ESM.doc]

**Additional file 1**

Within groups covariance matrix used in modeling:

| 1. Training |  |  |  |  |  |  |  |
| --- | --- | --- | --- | --- | --- | --- | --- |
| Activity | 1.16218 | 0.23774 | 2.0439 | 0.7759 | 1.3038 | 0.9596 | 2.0272 |
| 2. CII | 0.23774 | 0.42303 | 1.3715 | 1.4026 | 1.6571 | 0.6444 | 0.9867 |
| 3. Influence | 2.04395 | 1.37150 | 47.0269 | 15.9672 | 16.1293 | 9.4541 | 11.8521 |
| 4. Adaptability | 0.77591 | 1.40256 | 15.9672 | 26.7770 | 13.9754 | 3.8689 | 6.5267 |
| 5. Efficacy | 1.30385 | 1.65707 | 16.1293 | 13.9754 | 23.6488 | 6.9292 | 7.1342 |
| 6. Orgclimate | 0.95965 | 0.64440 | 9.4541 | 3.8689 | 6.9292 | 30.0493 | 27.3312 |
| 7. Dleadership | 2.02723 | 0.98671 | 11.8521 | 6.5267 | 7.1342 | 27.3312 | 55.4572 |

**Raw Data for subsample of** counselors who did training:

| Obs | accept | approp | adopt | prepadq | leader | CII | influence | CIIinfl |
| --- | --- | --- | --- | --- | --- | --- | --- | --- |
| 1 | 43.7500 | 45.7143 | 50 | 46.0000 | 42 | 4.55556 | 40.0000 | 1 |
| 2 | 45.0000 | 48.5714 | 50 | 40.0000 | 42 | 4.22222 | 30.0000 | 0 |
| 3 | 43.7500 | 47.1429 | 50 | 44.0000 | 38 | 3.11111 | 41.6667 | 0 |
| 4 | 45.0000 | 50.0000 | 50 | 48.0000 | 44 | 3.44444 | 33.3333 | 0 |
| 5 | 42.5000 | 47.1429 | 50 | 48.0000 | 40 | 4.22222 | 36.6667 | 1 |
| 6 | 45.0000 | 41.4286 | 50 | 46.0000 | 36 | 4.22222 | 33.3333 | 0 |
| 7 | 37.5000 | 31.4286 | 40 | 34.0000 | 42 | 2.77778 | 40.0000 | 0 |
| 8 | 41.2500 | 41.4286 | 50 | 44.0000 | 32 | 4.33333 | 41.6667 | 1 |
| 9 | 43.7500 | 38.5714 | 50 | 46.0000 | 44 | 3.88889 | 41.6667 | 1 |
| 10 | 40.0000 | 34.2857 | 40 | 42.0000 | 34 | 4.44444 | 45.0000 | 1 |
| 11 | 41.2500 | 41.4286 | 50 | 46.0000 | 40 | 3.66667 | 38.3333 | 1 |
| 12 | 41.2500 | 40.0000 | 50 | 40.0000 | 42 | 2.33333 | 50.0000 | 0 |
| 13 | 46.2500 | 47.1429 | 50 | 44.0000 | 38 | 4.11111 | 48.3333 | 1 |
| 14 | 41.4286 | 42.5000 | 50 | 33.3333 | 40 | 3.88889 | 50.0000 | 1 |
| 15 | 38.7500 | 47.1429 | 40 | 42.0000 | 38 | 3.22222 | 41.6667 | 0 |
| 16 | 43.7500 | 48.5714 | 50 | 50.0000 | 46 | 4.66667 | 48.3333 | 1 |
| 17 | 45.0000 | 47.1429 | 50 | 50.0000 | 44 | 4.66667 | 48.3333 | 1 |
| 18 | 43.7500 | 41.4286 | 40 | 38.0000 | 36 | 4.11111 | 45.0000 | 1 |
| 19 | 45.0000 | 48.5714 | 50 | 50.0000 | 30 | 4.22222 | 38.3333 | 1 |
| 20 | 37.5000 | 37.1429 | 40 | 40.0000 | 34 | 3.77778 | 30.0000 | 0 |
| 21 | 36.2500 | 35.7143 | 40 | 42.0000 | 46 | 2.88889 | 46.6667 | 0 |
| 22 | 40.0000 | 42.8571 | 30 | 36.0000 | 26 | 3.44444 | 46.6667 | 0 |
| 23 | 43.7500 | 40.0000 | 50 | 42.0000 | 38 | 3.55556 | 40.0000 | 1 |
| 24 | 45.0000 | 41.4286 | 50 | 44.0000 | 38 | 3.33333 | 40.0000 | 0 |
| 25 | 41.2500 | 45.7143 | 50 | 44.0000 | 40 | 3.55556 | 38.3333 | 1 |
| 26 | 42.5000 | 42.8571 | 40 | 42.0000 | 35 | 3.11111 | 40.0000 | 0 |
| 27 | 45.0000 | 41.4286 | 40 | 40.0000 | 34 | 2.66667 | 33.3333 | 0 |
| 28 | 41.2500 | 44.2857 | 40 | 40.0000 | 34 | 3.33333 | 50.0000 | 0 |
| 29 | 42.5000 | 41.4286 | 40 | 40.0000 | 24 | 4.66667 | 46.6667 | 1 |
| 30 | 42.5000 | 41.4286 | 50 | 42.0000 | 36 | 3.55556 | 35.0000 | 1 |
| 31 | 42.5000 | 41.4286 | 40 | 40.0000 | 38 | 4.00000 | 40.0000 | 1 |
| 32 | 45.0000 | 50.0000 | 50 | 50.0000 | 50 | 5.00000 | 41.6667 | 1 |
| 33 | 36.2500 | 37.1429 | 40 | 36.0000 | 34 | 4.00000 | 33.3333 | 0 |
| 34 | 32.5000 | 38.5714 | 20 | 38.0000 | 32 | 3.22222 | 38.3333 | 0 |
| 35 | 41.2500 | 40.0000 | 40 | 36.0000 | 32 | 3.22222 | 41.6667 | 0 |
| 36 | 45.0000 | 47.1429 | 50 | 42.0000 | 46 | 3.44444 | 46.6667 | 0 |
| 37 | 42.5000 | 38.5714 | 40 | 40.0000 | 40 | 3.44444 | 41.6667 | 0 |
| 38 | 40.0000 | 40.0000 | 50 | 42.0000 | 40 | 3.55556 | 33.3333 | 0 |
| 39 | 40.0000 | 38.5714 | 40 | 38.0000 | 40 | 3.22222 | 28.3333 | 0 |
| 40 | 42.5000 | 44.2857 | 50 | 44.0000 | 48 | 3.55556 | 41.6667 | 1 |
| 41 | 38.7500 | 45.7143 | 50 | 44.0000 | 42 | 3.00000 | 38.3333 | 0 |
| 42 | 36.2500 | 38.5714 | 40 | 40.0000 | 38 | 2.66667 | 40.0000 | 0 |
| 43 | 45.0000 | 47.1429 | 50 | 48.0000 | 32 | 3.88889 | 35.0000 | 1 |
| 44 | 43.7500 | 40.0000 | 40 | 38.0000 | 38 | 3.11111 | 36.6667 | 0 |
| 45 | 42.5000 | 47.1429 | 50 | 46.0000 | 44 | 4.55556 | 41.6667 | 1 |
| 46 | 41.2500 | 44.2857 | 50 | 42.0000 | 46 | 3.12500 | 35.0000 | 0 |
| 47 | 37.5000 | 38.5714 | 40 | 38.0000 | 40 | 2.77778 | 40.0000 | 0 |
| 48 | 40.0000 | 40.0000 | 40 | 40.0000 | 36 | 3.77778 | 35.0000 | 1 |
| 49 | 45.0000 | 50.0000 | 30 | 50.0000 | 48 | 3.00000 | 40.0000 | 0 |
| 50 | 43.7500 | 50.0000 | 50 | 44.0000 | 38 | 4.44444 | 40.0000 | 1 |
| 51 | 43.7500 | 47.1429 | 50 | 42.0000 | 38 | 4.22222 | 41.6667 | 1 |
| 52 | 46.2500 | 42.8571 | 50 | 42.0000 | 38 | 3.88889 | 38.3333 | 1 |
| 53 | 45.0000 | 40.0000 | 40 | 48.0000 | 38 | 4.11111 | 43.3333 | 1 |
| 54 | 42.5000 | 40.0000 | 50 | 42.0000 | 40 | 3.33333 | 40.0000 | 0 |
| 55 | 42.5000 | 41.4286 | 50 | 40.0000 | 44 | 3.66667 | 40.0000 | 1 |
| 56 | 43.7500 | 47.1429 | 50 | 46.0000 | 44 | 4.22222 | 41.6667 | 1 |
| 57 | 45.0000 | 41.4286 | 50 | 42.0000 | 40 | 3.66667 | 40.0000 | 1 |
| 58 | 36.2500 | 34.2857 | 40 | 32.0000 | 34 | 3.55556 | 46.6667 | 1 |
| 59 | 35.0000 | 32.8571 | 40 | 38.0000 | 40 | 2.66667 | 41.6667 | 0 |
| 60 | 42.5000 | 38.5714 | 50 | 42.0000 | 40 | 3.77778 | 36.6667 | 1 |
| 61 | 41.2500 | 41.4286 | 50 | 38.0000 | 40 | 3.77778 | 20.0000 | 0 |
| 62 | 43.7500 | 44.2857 | 40 | 48.0000 | 32 | 3.00000 | 26.6667 | 0 |
| 63 | 37.5000 | 37.1429 | 40 | 42.0000 | 42 | 3.44444 | 50.0000 | 0 |
| 64 | 46.2500 | 44.2857 | 50 | 46.0000 | 36 | 3.22222 | 43.3333 | 0 |
| 65 | 42.5000 | 42.8571 | 40 | 44.0000 | 30 | 3.22222 | 45.0000 | 0 |
| 66 | 45.0000 | 42.8571 | 50 | 44.0000 | 42 | 3.11111 | 41.6667 | 0 |
| 67 | 43.7500 | 34.2857 | 50 | 36.0000 | 40 | 3.77778 | 38.3333 | 1 |
| 68 | 43.7500 | 40.0000 | 50 | 42.0000 | 34 | 3.11111 | 35.0000 | 0 |
